# Correction: Amer et al. Assessing Patient Experience and Attitude: BSC-PATIENT Development, Translation, and Psychometric Evaluation—A Cross-Sectional Study. *Int. J. Environ. Res. Public Health* 2022, *19*, 7149

**DOI:** 10.3390/ijerph191912319

**Published:** 2022-09-28

**Authors:** Faten Amer, Sahar Hammoud, David Onchonga, Abdulsalam Alkaiyat, Abdulnaser Nour, Dóra Endrei, Imre Boncz

**Affiliations:** 1 Doctoral School of Health Sciences, Faculty of Health Sciences, University of Pécs, H-7621 Pécs, Hungary; 2 Institute for Health Insurance, Faculty of Health Sciences, University of Pécs, H-7621 Pécs, Hungary; 3 Division of Public Health, Faculty of Medicine and Health Sciences, An Najah National University, Nablus P.O. Box 7, Palestine; 4 Faculty of Economics and Social Sciences, An Najah National University, Nablus P.O. Box 7, Palestine; 5 National Laboratory for Human Reproduction, University of Pécs, H-7621 Pécs, Hungary

The authors wish to make the following corrections to this paper [1]:

## Text Correction

There was an error in the “Abstract” of the original publication. We mentioned that “The final best fit model in CFA comprised ten constructs with 34 items.”

A correction has been made to “The final best fit model in CFA comprised ten constructs with 36 items.”

There was an error in the “Conclusions” of the original publication. We mentioned that “It consists of 19 items assessing patient experience observations and 15 items assessing patient attitudes.”

A correction has been made to “It consists of 36 items; 21 items assessing patient experience observations and 15 items assessing patient attitudes.” 

## Error in Figure

In the original publication, there were mistakes in Figure 5 as published. The corrected Figure 5 appears below.

## Error in Table

In the original publication, there was a mistake in Table A3 as published. We missed two lines of data. The corrected Table A3 appears below.

## Figures and Tables

**Figure 5:**
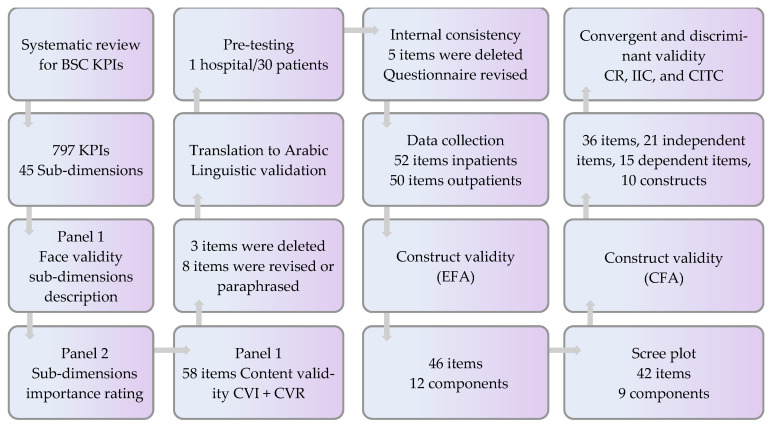
Flow chart for BSC-PATIENT development and psychometric validation.

## Appendix A

**Table A3:** Final resulted items.

Construct	Code	No.	Question
PT EXR	PCU4	Q1	This hospital distributes surveys to assess my satisfaction before discharge.
	PCU3	Q2	This hospital distributes surveys to assess my needs upon arrival to the hospital, admission, or during the stay.
	PLE11	Q3	This hospital follows up with me after the discharge.
	PCU5	Q4	My complaints are taken seriously into consideration and solved immediately at this hospital.
	PLE6	Q5	Staff trained me on infection precaution measures such as hand hygiene, cough etiquette, isolation rational, personal protective equipment, etc.
PR EXR	PFI2	Q6	I pay a reasonable price for the other medical services (laboratory, radiology, etc.) at this hospital.
	PFI3	Q7	I pay a reasonable price for the medications at this hospital.
	PFI1	Q8	I pay a reasonable price for the medical consultation at this hospital.
BUILENV EXR	PEN13	Q9	There is a sufficient number of chairs in the waiting area.
	PEN12	Q10	The hospital has clean departments, corridors, rooms, bathrooms.
BUILCAP EXR	PEN14	Q11	The capacity of departments at this hospital including (ER, ICU, waiting room, etc.) is sufficient enough.
	PEN11	Q12	This hospital has new building infrastructure (walls, ceiling, bathrooms, etc.).
ACC EXR	PEN4	Q13	The accessibility to this hospital is easy by either public transportation or my car.
	PEN5	Q14	The accessibility to this hospital in an emergency is easy.
INFO EXR	PLE4	Q15	Information provided to me to be used after discharge is sufficient (medication and side effects, health condition, etc.).
	PLE3	Q16	Oral and written information provided to me or my family during my hospital experience is sufficient.
	PLE2	Q17	Information and guidance provided at admission or the first visit are sufficient.
SERV	PEN8	Q18	Female doctors are available at this hospital.
	PIN12	Q19	There are a variety of departments at this hospital.
	PIN18	Q20	Services at night, vacations, and weekends are available at this hospital.
	PIN15	Q21	There are a variety of specialties at this hospital.
BSCP ATT	SAT3	Q22	I will recommend this hospital to my family and friends.
	PIN1	Q23	I believe I receive an accurate medical examination at this hospital.
	PEN3	Q24	I believe this hospital offers me better treatment than the other Palestinian hospitals.
	SAT1	Q25	My overall satisfaction with this hospital’s performance is high.
	PEN1	Q26	I believe this hospital has a high cure rate.
	SAT2	Q27	I will choose this hospital again when I need a medical consultation.
	PLE1	Q28	I believe the staff at this hospital are competent, knowledgeable, updated, and skilled.
	PIN16	Q29	The services provided to me at this hospital have high quality.
	PIN14	Q30	I believe the medications prescribed to me at this hospital have good quality and efficacy.
COMP IMAGE	PIN6	Q31	There is a probability for postoperative bacterial infection at this hospital
	PIN5	Q32	There is a probability for case referral to another hospital
	PIN4	Q33	There is a probability for case readmission at the same hospital
TECH IMAGE	PLE9	Q34	This hospital use technology to link my prescriptions and tests with pharmacy and labs.
	PLE10	Q35	This hospital use technology for saving my records.
	PLE8	Q36	I believe this hospital uses the newest technology and devices for diagnosing and treating patients.

The authors apologize for any inconvenience caused and state that the scientific conclusions are unaffected. The original publication has also been updated.
